# Prognostic Machine Learning Models for First-Year Mortality in Incident Hemodialysis Patients: Development and Validation Study

**DOI:** 10.2196/20578

**Published:** 2020-10-29

**Authors:** Kaixiang Sheng, Ping Zhang, Xi Yao, Jiawei Li, Yongchun He, Jianghua Chen

**Affiliations:** 1 Kidney Disease Center The First Affiliated Hospital, Zhejiang University School of Medicine Hangzhou China

**Keywords:** machine learning, hemodialysis, XGBoost, prediction model

## Abstract

**Background:**

The first-year survival rate among patients undergoing hemodialysis remains poor. Current mortality risk scores for patients undergoing hemodialysis employ regression techniques and have limited applicability and robustness.

**Objective:**

We aimed to develop a machine learning model utilizing clinical factors to predict first-year mortality in patients undergoing hemodialysis that could assist physicians in classifying high-risk patients.

**Methods:**

Training and testing cohorts consisted of 5351 patients from a single center and 5828 patients from 97 renal centers undergoing hemodialysis (incident only). The outcome was all-cause mortality during the first year of dialysis. Extreme gradient boosting was used for algorithm training and validation. Two models were established based on the data obtained at dialysis initiation (model 1) and data 0-3 months after dialysis initiation (model 2), and 10-fold cross-validation was applied to each model. The area under the curve (AUC), sensitivity (recall), specificity, precision, balanced accuracy, and F1 score were used to assess the predictive ability of the models.

**Results:**

In the training and testing cohorts, 585 (10.93%) and 764 (13.11%) patients, respectively, died during the first-year follow-up. Of 42 candidate features, the 15 most important features were selected. The performance of model 1 (AUC 0.83, 95% CI 0.78-0.84) was similar to that of model 2 (AUC 0.85, 95% CI 0.81-0.86).

**Conclusions:**

We developed and validated 2 machine learning models to predict first-year mortality in patients undergoing hemodialysis. Both models could be used to stratify high-risk patients at the early stages of dialysis.

## Introduction

### Background

The overall prevalence of chronic kidney disease is 10.8% in China and 15% in the United States, which has brought significant economic, social, and medical burdens on patients and society [1-3]. According to the United States Renal Data System, there are approximately 120,000 patients with end-stage renal disease starting chronic renal replacement therapy every year [2]. However, survival among incident hemodialysis patients remains poor, especially in the first year of the initiation of dialysis [4,5].

End-stage renal disease is a complex disease state with multiple associated comorbidities. Patients initiating hemodialysis often have acute complications, and some of them suffer from major comorbid conditions that are associated with poor short-term prognoses [6]. It is essential to stratify the risk of mortality according to clinical and laboratory findings of patients undergoing hemodialysis; therefore, the identification of patients undergoing hemodialysis who are at high risk of first-year mortality is of great clinical significance. It can inform patients of their survival prognosis in the early stages of dialysis and allow clinicians to make targeted intervention strategies to improve first-year outcomes. Previous studies [7-11] have identified many risk factors for early dialysis mortality, such as old age, chronic heart failure, catheter use, low albumin, low hemoglobin, and high estimated glomerular filtration rate at dialysis initiation. However, because of the heterogeneity of primary disorders and broad comorbidities, these risk factors are not enough to be used for conclusive decision making. In recent years, a number of clinical risk models have been developed to predict early mortality in the dialysis population, and most are based on linear models (logistic or Cox model) [12-16]. The performances of these models were not good enough in either the original population or the external validation—area under the curve (AUC) of these models ranged from 0.710 to 0.752 [17]. In addition, no study compared models based on predialysis data with models based on data after dialysis.

In recent years, machine learning has been proven to be a very powerful method by researchers in medical fields [18-21]. Machine learning is useful in identifying the most important factors and for developing predictive models with the best performance. A recent study [22] reported on a random forest machine learning model used to predict first-year survival of incident hemodialysis patients. The model’s AUC was 0.749 (95% CI 0.742-0.755), which was superior to those of traditional risk prediction models; however, this is not accurate enough for clinical application.

### Objective

Therefore, in this study, we sought to develop and validate sufficiently accurate models based on machine learning techniques, utilizing readily available clinical factors to predict first-year mortality in incident dialysis patients.

## Methods

### Study Design

**Figure 1:**
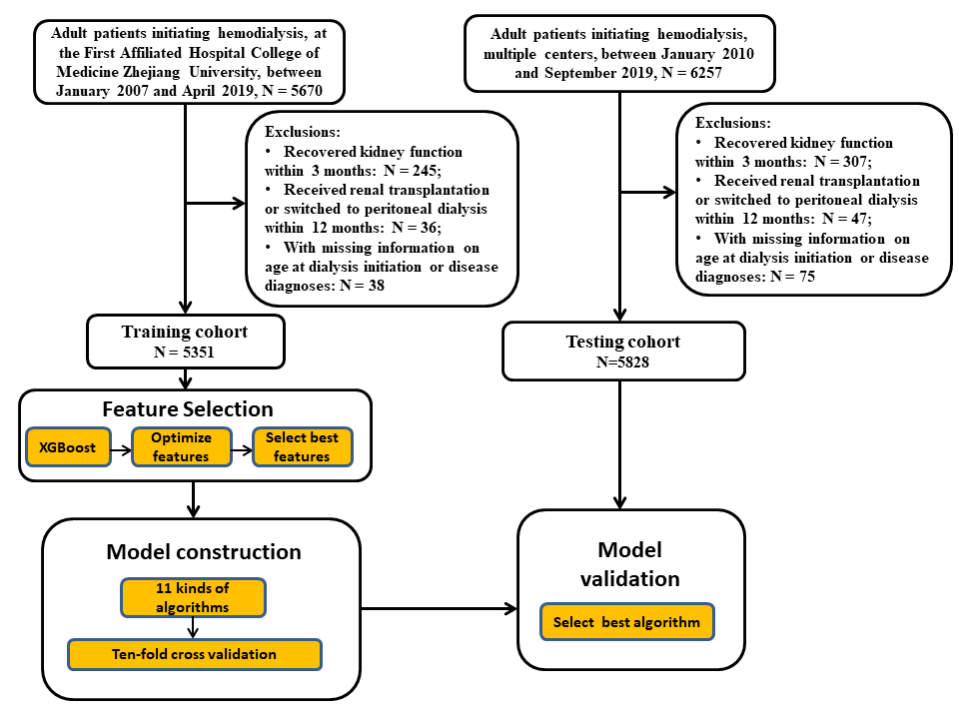
A workflow to develop the prediction models for first-year mortality in incident hemodialysis patients. XGBoost: extreme gradient boosting.

This study retrospectively collected data from Zhejiang Dialysis System. Zhejiang Dialysis System is a database of hemodialysis and peritoneal dialysis patients in East China. Training data were retrieved from the First Affiliated Hospital College of Medicine Zhejiang University between January 2007 and April 2019 (Figure 1). Testing data were collected from 97 renal centers between January 2010 and August 2018 for external validation (Figure 1). All follow-up data were updated to August 2019.

Adult patients (aged ≥18 years) with end-stage renal disease and with follow-up exceeding 12 months who started maintenance hemodialysis were included. Patients who died within 12 months of follow-up were also included.

The exclusion criteria were as follows: patients with a history of previous renal replacement therapy, patients whose kidney function recovered within 3 months, patients who received renal transplantation or switched to peritoneal dialysis within 12 months after dialysis initiation. We also excluded patients with missing information on disease diagnoses or age at dialysis initiation.

This study followed the tenets of the Declaration of Helsinki and was approved by the ethics committee of the First Affiliated Hospital of Zhejiang University (IIT20200088A) in Hangzhou, China. Written informed consent was obtained from each participant.

### Outcome and Predictors

The outcome of this study was all-cause mortality during the first year of dialysis. Outcome status and potential candidate variables for the prediction tool, including demographic information, disease diagnoses, comorbidities, and laboratory test results, were obtained from the Zhejiang Dialysis System.

Demographic information and type of vascular access were collected at the start of dialysis. Disease diagnoses, comorbid information, and laboratory test results were collected 0-3 months after dialysis initiation. The most recent serum creatinine measurements prior to the index date were used to estimate the glomerular filtration rate using the Chronic Kidney Disease Epidemiology Collaboration equation [23].

A total of 42 variables were included as candidate features based on review of relevant literature and clinical experience. Only BMI and ferritin had missing data, and both instances of missing data were less than 6% (Table 1).

**Table 1:** Baseline characteristics of the training and testing cohorts.

Characteristics		At dialysis initiation				0-3 months			
		Training cohort (n=5351)		Testing cohort (n=5828)		Training cohort (n=4425)		Testing cohort (n=3729)	
**Sex, n (%)**									
	Male		3295 (61.58)		3524 (60.47)		2744 (62.01)		2264 (60.71)
	Female		2056 (38.42)		2304 (39.53)		1681 (37.99)		1465 (39.29)
Body mass index (kg/m^2^), mean (SD)^a^		22.09 (3.29)		21.73 (3.07)		22.19 (3.39)		21.83 (3.04)	
Age at dialysis initiation (years), mean (SD)		51.67 (16.48)		62.53 (16.20)		52.61 (16.59)		62.45 (15.9)	
Systolic pressure (mmHg), mean (SD)		137.49 (22.93)		146.18 (24.58)		138.52 (23.15)		146.33 (24.68)	
Diastolic pressure (mmHg), mean (SD)		77.76 (12.26)		78.95 (15.52)		80.45 (12.15)		79.02 (15.45)	
**Chronic kidney disease etiology, n (%)**									
	Chronic glomerulonephritis		2823 (52.76)		3015 (51.73)		2445 (55.25)		2064 (55.35)
	Diabetic nephropathy		1120 (20.93)		1191 (20.44)		895 (20.23)		818 (21.94)
	Hypertensive nephropathy		262 (4.90)		557 (9.56)		218 (4.93)		370 (9.92)
	Lupus nephritis		68 (1.27)		50 (0.86)		57 (1.29)		29 (0.78)
	ANCA-associated^b^ vasculitis		57 (1.07)		64 (1.10)		53 (1.20)		33 (0.88)
	Gouty nephropathy		32 (0.60)		125 (2.14)		26 (0.59)		72 (1.93)
	Polycystic kidney disease		286 (5.34)		214 (3.67)		220 (4.97)		150 (4.02)
	Other		703 (13.14)		612 (11.07)		511 (11.54)		204 (5)
**Comorbid conditions, n (%)**									
	Cirrhosis		86 (1.61)		90 (1.54)		81 (1.83)		60 (1.61)
	Multiple myeloma		46 (0.86)		90 (1.54)		46 (1.04)		51 (1.37)
	Atrial fibrillation		108 (2.02)		109 (1.87)		85 (1.92)		72 (1.93)
	Congestive heart failure		969 (18.11)		999 (17.14)		794 (17.94)		605 (16.22)
	Ischemic heart disease		1476 (27.58)		1578 (27.08)		1206 (27.25)		983 (26.36)
	Metastatic cancer		86 (1.61)		91 (1.56)		74 (1.67)		38 (1.02)
	Lymphoma		7 (0.13)		7 (0.12)		6 (0.14)		1 (0.03)
	Chronic obstructive pulmonary disease		241 (4.50)		165 (2.83)		169 (3.82)		78 (2.09)
	Cerebrovascular disease		322 (6.02)		411 (7.05)		244 (5.51)		271 (7.27)
**Laboratory data**									
	Leukocyte (10^9^/L), mean (SD)		7.32 (2.95)		7.71 (3.79)		7.40 (3.09)		6.90 (3.22)
	Neutrophil (10^9^/L), mean (SD)		5.23 (2.68)		5.06 (3.32)		5.36 (2.78)		4.22 (2.57
	Hemoglobin (g/L), mean (SD)		94.82 (23.30)		83.09 (19.12)		91.05 (21.68)		86.50 (14.67)
	Platelet (10^9^/L), mean (SD)		193.28 (93.47)		182.47 (83.70)		190.84 (88.13)		184.36 (71.39)
	Albumin (g/L), mean (SD)		36.01 (6.75)		33.27 (5.99)		36.80 (6.59)		33.98 (5.54)
	Phosphorus (mmol/L), mean (SD)		1.81 (0.62)		1.70 (0.66)		1.66 (0.52)		1.54 (0.50)
	Calcium (mmol/L), mean (SD)		2.15 (0.28)		2.02 (0.30)		2.14 (0.22)		2.08 (0.23)
	Potassium (mmol/L)		4.87 (1.11)		4.52 (0.91)		4.76 (0.96)		4.42 (0.69)
	Parathyroid hormone (pg/ml), mean (SD)		334.71 (292.07)		246.95 (193.61)		315.98 (291.84)		241.26 (206.48)
	Creatinine (μmol/L), mean (SD)		807.11 (352.04)		718.84 (336.47)		755.28 (315.95)		661.5 (268.48)
	Urea nitrogen (mmol/L), mean (SD)		22.65 (12.07)		23.61 (11.77)		19.87 (8.72)		20.01 (8.13)
	Uric acid (μmol/L), mean (SD)		436.84 (147.54)		450.27 (157.44)		392.87 (126.48)		402.19 (113.46)
	C-reactive protein, mean (SD)		40.84 (44.09)		25.65 (44.46)		18.52 (35.01)		20.23 (31.22)
	Cholesterol (mmol/L), mean (SD)		4.34 (1.30)		4.30 (1.42)		4.27 (1.23)		4.34 (1.25)
	Triglycerides (mmol/L), mean (SD)		1.56 (1.00)		1.60 (1.03)		1.58 (0.96)		1.63 (0.97)
	High-density lipoprotein, (mmol/L), mean (SD)		1.14 (0.42)		1.11 (0.43)		1.12 (0.39)		1.15 (0.38)
	Low-density lipoprotein (mmol/L), mean (SD)		2.36 (1.10)		2.37 (1.02		2.31 (1.04)		2.35 (0.92)
	Very low-density lipoprotein (mmol/L), mean (SD)		1.65 (1.55)		2.11 (1.35)		1.63 (1.54)		1.60 (0.93)
	Ferritin (ng/mL), mean (SD)^c^		174.59 (126.34)		328.25 (295.78)		144.34 (144.87)		305.42 (278.73)
	eGFR^d^ (mL/min/1.73m^2^), mean (SD)		6.75 (3.79)		7.28 (3.93)		7.23 (3.85)		7.58 (3.44)
**Vascular access at dialysis initiation, n (%)**									
	Nontunneled catheter		3295 (61.58)		3388 (58.13)		2495 (56.38)		1893 (50.76)
	Tunneled catheter		1068 (19.96)		1266 (21.72)		1005 (22.71)		938 (25.15)
	Fistula or graft		988 (18.46)		1174 (20.14)		925 (20.90)		898 (24.08)
Death at 1-year follow-up, n (%)		585 (10.93)		764 (13.11)		437 (9.88)		477 (12.79)	

^a^The missing rates of body mass index in the 4 cohorts were 270 (5.04%), 298 (5.11%), 210 (4.74%), and 168 (4.50%), respectively.

^b^ANCA: antineutrophil cytoplasmic antibody.

^c^The missing rates of ferritin in the 4 cohorts were 0.36%, 3.00%, 0.36%, and 2.13%, respectively.

^d^eGFR: estimated glomerular filtration rate.

### Data Preprocessing

Before the baseline model was developed, missing data were imputed with the mean value for continuous variables and the mode value for categorical variables. By using one-hot encoding, all categorical features were transformed into numerical features. Box-Cox transformation was performed to normalize numerical features that were highly skewed [24].

### Algorithm Development and Validation

An extreme gradient boosting machine learning algorithm was employed to build a model to predict the correlation between features and the outcome. Extreme gradient boosting is an integrated learning algorithm based on gradient boosted decision trees [25]. Using the Gini impurity index [26], we estimated the feature importance scores of candidate features after going through the training process. The feature importance scores showed how valuable each feature was in the construction of the boosted decision trees within the model.

The extreme gradient boosting algorithm was employed because (1) it has high efficiency and accuracy, (2) it can prevent overfitting via regularization, (3) it provides feature importance, and (4) it allows the use of a wide variety of computing environments.

Other popular machine learning algorithms—adaptive boosting, light gradient boosting machine, logistic regression, linear discriminant analysis, random forest, extra trees, gradient boosting, multiple layers perception, k-nearest neighbor, and decision trees—were compared with extreme gradient boosting.

We developed 2 models that were based on the data obtained at dialysis initiation (model 1) and data 0-3 months after dialysis initiation (model 2); 10-fold cross-validation was used to avoid overfitting and to validate each model [27]. We measured AUC, sensitivity (recall), specificity, precision, balanced accuracy, and F1 score to assess the predictive ability of each model. The balanced accuracy was calculated as follows: balanced accuracy = (sensitivity + specificity) / 2. The F1 score were calculated as follows: F1 score = (2 × precision × recall) / (precision + recall). Shapley additive explanation (SHAP) values were used to measure the marginal contribution of each feature to the models [28].

## Results

### Demographic and Clinical Characteristics

The demographic and clinical characteristics of the training and testing cohorts indicated that most characteristics were similarly distributed (Table 1). All patients were Chinese. The mean ages at dialysis initiation were 51.67 years (SD 16.48) in the training cohort and 62.53 years (SD 16.20) in the testing cohort; 61.58% of the patients (3295/5351) in the training cohort and 60.47% of the patients (3524/5828) in the testing cohort were men; out of 5351 patients, 585 (10.93%) deaths were reported in the training cohort, and out of 5828 patients, 764 (13.11%) deaths were reported in the testing cohort.

### Model Performance

The ranks of features selected after training the extreme gradient boosting models are shown in Multimedia Appendix 1 and Multimedia Appendix 2. The same 15 most important features were chosen for both model 1 and model 2: age at dialysis initiation, vascular access, metastatic cancer, diabetic nephropathy, congestive heart failure, ischemic heart disease, cerebrovascular disease, albumin, hemoglobin, neutrophil, C-reactive protein, creatinine, estimated glomerular filtration rate, systolic blood pressure, and BMI.

**Figure 2:**
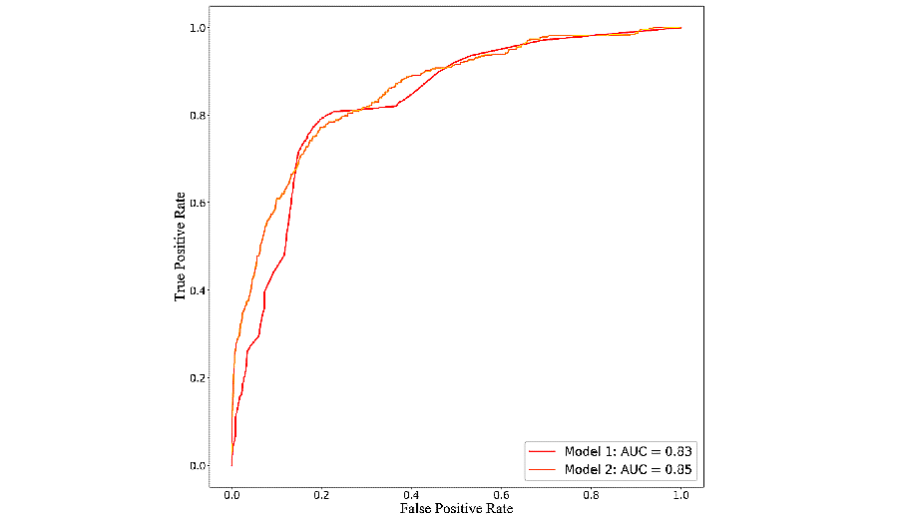
Receiver-operating characteristic curves of model 1 and model 2. AUC: the area under the curve.

Among the 11 algorithms applied (Table 2), the extreme gradient boosting algorithm had the best generalized performance for both model 1 (AUC 0.83, 95% CI 0.78-0.84; balanced accuracy 84.52%; F1 score 0.75) and model 2 (AUC 0.85, 95% CI 0.81-0.86, balanced accuracy 89.21%, F1 score 0.78). As shown in Figure 2, the receiver operating characteristic curves of both models were similar.

**Figure 3:**
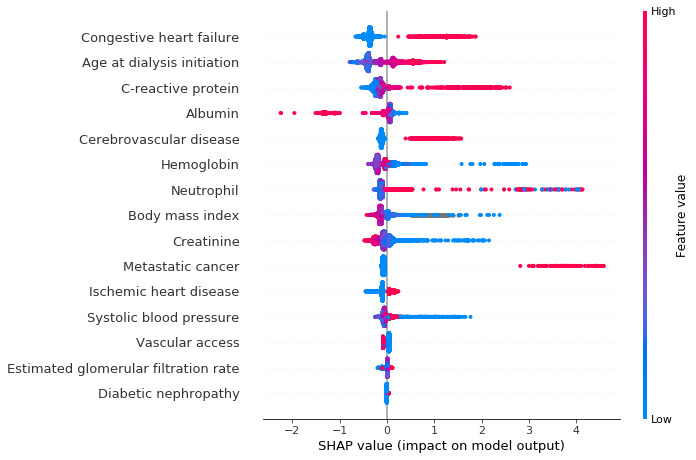
SHAP values illustrating how features contribute to model 1. Blue shows a negative contribution, and red shows a positive contribution. SHAP: Shapley additive explanation.

**Figure 4:**
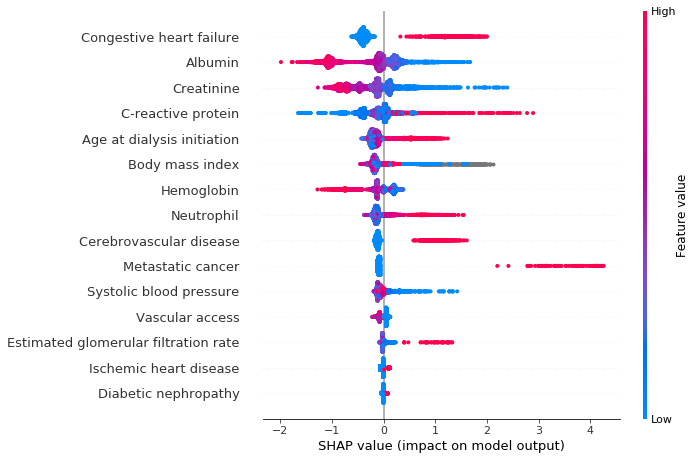
SHAP values illustrating how features contribute to model 2. Blue shows a negative contribution, and red shows a positive contribution. SHAP: Shapley additive explanation.

**Figure 5:**
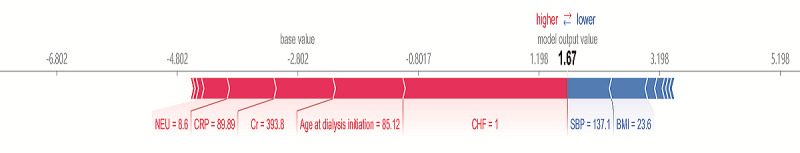
The SHAP value for a single data sample. BMI: body mass index, CHF: congestive heart failure, CRP: C-reactive protein, Cr: creatinine, NEU: neutrophil, SBP: systolic blood pressure.

SHAP value results are shown in Figure 3 (model 1) and Figure 4 (model 2). Each point represents a data sample for the feature. History of congestive heart failure, albumin level, C-reactive protein level, and age at dialysis initiation were the most important factors affecting the prediction for first-year mortality in both model 1 and model 2. Figure 5 shows an example using model 2 that shows how features contribute to the probability for a single participant. This participant had a history of congestive heart failure, low creatinine level, a high C-reactive protein level, high neutrophil count, and old age at dialysis initiation, which contributed to a higher probability of mortality in the first year, although he had normal BMI and slightly high systolic blood pressure levels.

**Table 2:** Performance of different algorithms trained on the testing data set.

Models		Precision, %	Sensitivity, %	Specificity, %	F1 score	Balanced accuracy, %	AUC^a^ (95% CI)	Accuracy, %
**Model 1**								
	Adaptive boosting	43.34	55.37	89.29	0.4862	72.33	0.81 (0.77-0.82)	84.92
	Decision tree	68.61	35.47	97.55	0.4676	66.51	0.78 (0.76-0.80)	89.41
	Extra trees	78.56	59.95	97.53	0.6800	78.74	0.83 (0.77-0.83)	92.60
	Gradient boosting	52.58	49.35	93.29	0.5091	71.32	0.82 (0.77-0.83)	87.53
	k-nearest neighbor	47.32	50.92	91.45	0.4905	71.18	0.76 (0.76-0.84)	86.14
	Linear discriminant analysis	14.02	82.46	23.74	0.2397	53.10	0.75 (0.74-0.84)	31.43
	Light gradient boosting	91.76	62.70	99.15	0.7449	80.92	0.82 (0.77-0.83)	94.37
	Logistic regression	14.16	85.47	21.84	0.2430	53.66	0.68 (0.68-0.85)	30.18
	Multiple layers perception	16.64	78.80	40.44	0.2748	59.62	0.80 (0.68-0.85)	45.47
	Random forest	90.62	40.45	99.37	0.5593	69.91	0.81 (0.78-0.83)	91.64
	Extreme gradient boosting	79.34	71.86	97.18	0.7541	84.52	0.83 (0.78-0.84)	93.86
**Model 2**								
	Adaptive boosting	61.83	72.33	93.45	0.6667	82.89	0.83 (0.80-0.84)	90.75
	Decision tree	78.50	63.52	97.45	0.7022	80.48	0.81 (0.80-0.82)	93.11
	Extra trees	74.48	60.59	96.96	0.6682	78.77	0.84 (0.80-0.85)	92.30
	Gradient boosting	83.08	67.92	97.97	0.7474	82.95	0.84 (0.82-0.85)	94.13
	k-nearest neighbor	87.37	52.20	98.89	0.6535	75.55	0.82 (0.81-0.86)	92.92
	Linear discriminant analysis	16.33	82.81	37.76	0.2728	60.29	0.76 ()0.76-0.86	43.52
	Light gradient boosting	77.97	75.68	96.86	0.7681	86.27	0.85 (0.80-0.85)	94.15
	Logistic regression	16.12	81.76	37.58	0.2692	59.67	0.73 (0.73-0.86)	43.23
	Multiple layers perception	16.19	80.08	39.21	0.2694	59.65	0.71 (0.71-0.86)	44.44
	Random forest	66.67	70.02	94.86	0.6830	82.44	0.82 (0.80-0.85)	91.69
	Extreme gradient boosting	78.95	78.62	96.92	0.7878	87.77	0.85 (0.81-0.86)	94.58

^a^AUC: area under the curve.

## Discussion

### Principal Findings

In this study, by implementing advanced machine learning techniques, we developed and validated 2 clinical risk prediction models for first-year mortality in incident hemodialysis patients. The 2 extreme gradient boosting models were established based on the data available at dialysis initiation and data from 0-3 months after dialysis initiation. The performance of model 1 (AUC 0.83) was similar to that of model 2 (AUC 0.85), suggesting that we can predict first-year mortality in patients undergoing hemodialysis at dialysis initiation.

Mortality for patients undergoing hemodialysis during the first year of dialysis initiation is high [4]. Therefore, early and precise individualized risk estimates are required for clinical decision making. Traditional strategies for building prediction models have contributed to quality improvement and decision support. Nevertheless, these models have some limitations that may lead to missing important predictors and relationships. Our prediction models (model 1: AUC 0.83, model 2: AUC 0.85), compared with previous models (AUC 0.710-0.752) [12-17], were more accurate in stratifying the risk of first-year mortality for patients undergoing hemodialysis. Our prediction models had several unique and important characteristics. First, many clinical features have been reported for the prediction of first-year mortality in incident hemodialysis patients; some of these features are interact with each other. Traditional prediction models do not account for interactions between input features. By using extreme gradient boosting, we selected the 15 most important features from 42 candidate features, and then combined them nonlinearly. Second, missing data and data noise are inevitable in clinical data collected from the real world, which is a complex problem for traditional strategies. Machine learning techniques can deal with missing data and data noise automatically to improve model performance. Third, relationships between data may change over time because of improvements in treatment and changing populations. For example, the rates of diabetic nephropathy and cardiovascular disease have been increasing yearly [1,2]. Traditional prediction models are always nonrenewable. Machine learning allows for continual updating of the model to incorporate new data and capture changes in the relationships between features. Finally, compared with traditional predictive models, machine learning models are more complex and harder to interpret; it is not easy to determine how these models make decisions. Therefore, we used SHAP values to interpret the models in this study. SHAP values for a single patient can help physicians evaluate prognosis and make individualized treatment regimens.

Previous studies [8,15,29] have used data from distinct time periods. Floege et al [15], by using 90- to 180-day baseline and 0- to 90-day baseline data for the prediction of first-year mortality, revealed that 2 Cox regression models had similar performances. Some studies [8,29] used data obtained at dialysis initiation to predict the 3- to 6- month mortality of patients undergoing hemodialysis. Akbilgic et al [17] developed a random forest model based on 49 predialysis patient features (AUC 0.75, 95% CI 0.74-0.76); however, it may be not feasible for all users because too many features are needed. Our models were based on 15 features that are easily available for clinicians. The performance of model 1 was satisfactory, suggesting that model 1 can be used to classify high-risk patients at the early stage of dialysis. The first-year mortality risk of dialysis patients may be reduced by personalized and targeted preventive therapies.

### Limitations and Future Work

Despite the promising prospects demonstrated by our study, it had some limitations. First, our training data were based on retrospective data generated from a single center. Therefore, a possible center effect cannot be excluded. Second, although no restriction was placed on ethnicity, all patients included were Chinese. The primary disease of end-stage renal disease and cardiovascular conditions of patients undergoing hemodialysis in China differ from those of patients undergoing hemodialysis in other regions [2,30]. Thus, the applicability of our models to other ethnic groups and regions needs to be confirmed. Third, we only assessed 1-year mortality, whereas long-term mortality is also important [31]. Therefore, we plan to establish a model to predict 2-year and 5-year mortality in future studies. Finally, therapeutic intervention data, such as dialysis dose and frequency, were not used in this study because therapeutic interventions were not always fixed until 1-2 months after dialysis initiation, and therapeutic interventions in patients varied. We also plan to display the prediction models on the website of the Zhejiang Dialysis Quality Control Center and as a mobile app for better application.

### Conclusions

To accurately predict first-year mortality in incident hemodialysis patients, we developed and validated 2 machine learning models based on data available at dialysis initiation and data 0-3 months after dialysis initiation. The overall diagnostic performances of the 2 models were similar. We hope our models may assist clinicians in stratifying the risk of mortality at the early stages of dialysis. Our models need to be evaluated in data sets of patients undergoing hemodialysis from other ethnic groups and regions before implementation in clinical practice. For future research, long-term mortality predictions for patients undergoing incident dialysis will be addressed.

## Appendix

Multimedia Appendix 1 Importance rankings of 42 features based on data at dialysis initiation.

Multimedia Appendix 2 Importance ranking of 42 features based on data 0-3 months after dialysis initiation.

## Abbreviations

AUC: area under the curve
BMI: body mass index
SHAP: Shapley additive explanation
