# Supplementary material for: Prognostic Machine Learning Models for First-Year Mortality in Incident Hemodialysis Patients: Development and Validation Study
Source: JMIR Med Inform. 2020 Oct 29;8(10):e20578. doi: 10.2196/20578 (PMC7661257; doi:10.2196/20578)

**Multimedia Appendix 2:** The feature importance ranking of 42 features based on data between 0-3 months after dialysis initiation.


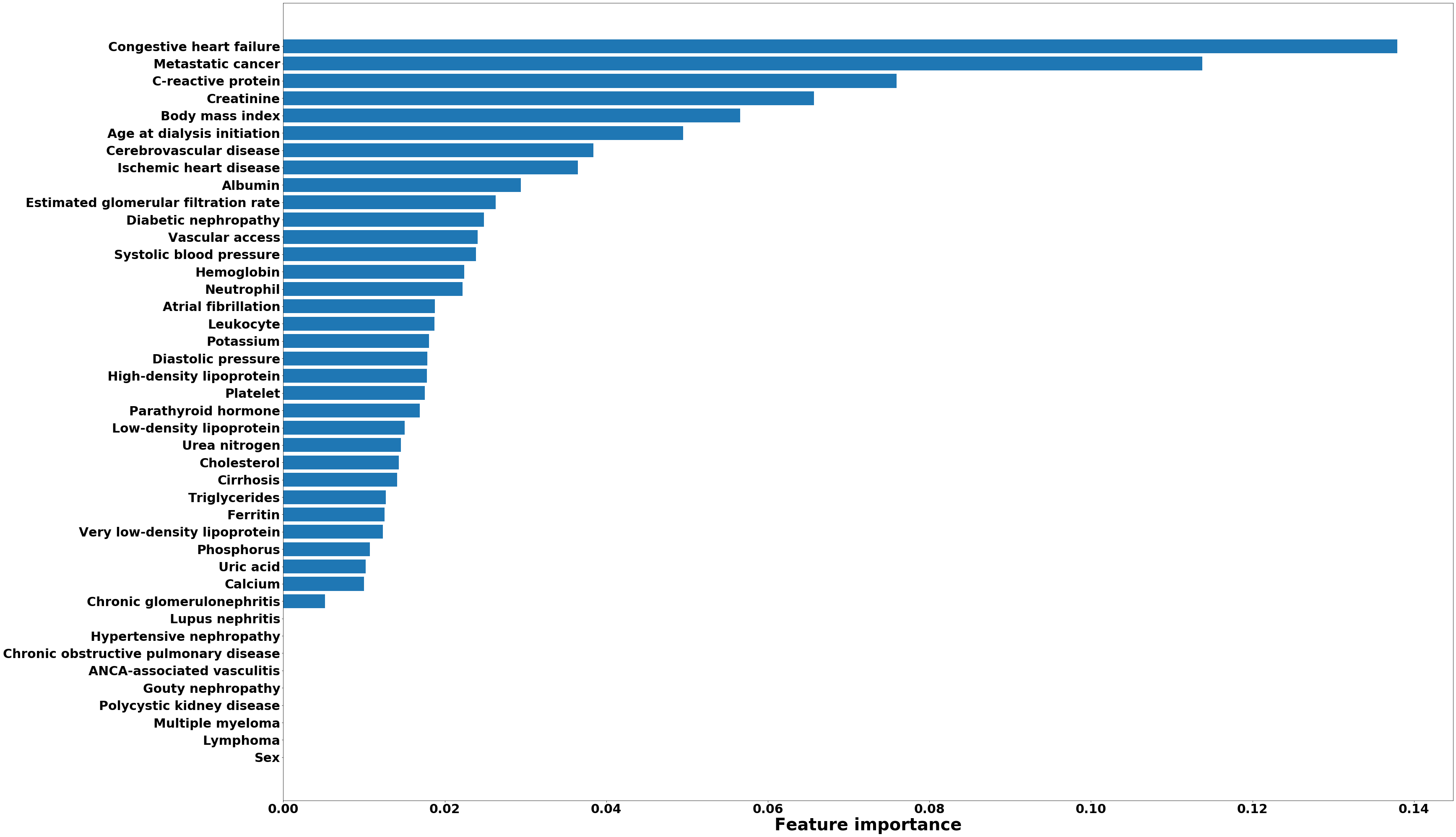

Supplement: Multimedia Appendix 2 [file medinform_v8i10e20578_app2.doc]
